# Early cysteine-dependent inactivation of 26S proteasomes does not involve particle disassembly

**DOI:** 10.1016/j.redox.2018.02.016

**Published:** 2018-02-22

**Authors:** Martín Hugo, Ioanna Korovila, Markus Köhler, Carlos García-García, J. Daniel Cabrera-García, Anabel Marina, Antonio Martínez-Ruiz, Tilman Grune

**Affiliations:** a Department of Molecular Toxicology, German Institute of Human Nutrition Potsdam-Rehbruecke (DIfE), 14558 Nuthetal, Germany; b Servicio de Proteómica, Centro de Biología Molecular “Severo Ochoa (CBSMO), Consejo Superior de Investigaciones Científicas (CSIC) – UAM, E-28049 Madrid, Spain; c Servicio de Inmunología, Hospital Universitario de La Princesa, Instituto de Investigación Sanitaria Princesa (IIS-IP), E-28006 Madrid, Spain; d Centro de Investigación Biomédica en Red de Enfermedades Cardiovasculares (CIBERCV), Spain; e German Center for Diabetes Research (DZD), 85764 München-Neuherberg, Germany; f German Center for Cardiovascular Research (DZHK), 10117 Berlin, Germany; g NutriAct-Competence Cluster Nutrition Research Berlin-Potsdam, Nuthetal 14458, Germany

## Abstract

Under oxidative stress 26S proteasomes suffer reversible disassembly into its 20S and 19S subunits, a process mediated by HSP70. This inhibits the degradation of polyubiquitinated proteins by the 26S proteasome and allows the degradation of oxidized proteins by a free 20S proteasome. Low fluxes of antimycin A-stimulated ROS production caused dimerization of mitochondrial peroxiredoxin 3 and cytosolic peroxiredoxin 2, but not peroxiredoxin overoxidation and overall oxidation of cellular protein thiols. This moderate redox imbalance was sufficient to inhibit the ATP stimulation of 26S proteasome activity. This process was dependent on reversible cysteine oxidation. Moreover, our results show that this early inhibition of ATP stimulation occurs previous to particle disassembly, indicating an intermediate step during the redox regulation of the 26S proteasome with special relevance under redox signaling rather than oxidative stress conditions.

## Highlights

• The 26S proteasome is sensitive to inhibition by cysteine oxidation during moderate mitochondrial ROS production.
• Early cysteine-dependent 26S proteasome inactivation occurs before particle disassembly.
• The 20S proteasomal activity remains constant under such moderate redox shifts.

## 1 Introduction

The ubiquitin-proteasomal system (UPS) is a complex, multi enzymatic pathway mainly responsible for the maintenance of cellular protein homeostasis. The so-called 20S proteasome is able to degrade oxidized, damaged and misfolded proteins while the 26S proteasome is able to degrade natively folded and functional proteins that have been tagged with ubiquitin, and its activity requires ATP hydrolysis [1]. The 26S proteasome consists of a core 20S proteasome that binds one or two 19S regulators [2], [3]. The abundance of the different particles highly varies between different cell types and organs [4]. The 19S regulatory particle itself contains ATP- and non-ATP-binding subunits, which are supposed to be responsible for a correct arrangement and movement of the protein into the proteasome and thus the rate of degradation [3], [5]. 19S binding increases proteolytic activity and enables the proteasome to degrade substrate proteins that have been labeled by a complex enzymatic machinery via the attachment of a chain of ubiquitin molecules [6].

The ubiquitin-proteasome system is known to be susceptible to oxidative stress. The oxidative inactivation of ubiquitin activating and conjugating enzymes could function as a mechanism to stop ATP-dependent proteolysis by the 26S proteasome and protein poly-ubiquitination [7]. Poly-ubiquitin accumulates during the oxidative stress response, and this has been recently attributed to oxidative inactivation of deubiquitinases in yeast [8]. Regarding the proteasome itself, the ATP-dependent 26S activity is highly susceptible to oxidative inactivation [9]. Contrary, the 20S proteasome is not only stable, but can also be activated by oxidation, especially by glutathionylation, leading to opening of the gate and increase in proteolytic activity [10], which allows the degradation of oxidized proteins [9]. Under acute oxidative stress, the inactivation of the 26S proteasome has been attributed to particle disassembly into its 20S and 19S particles, a process known to be dependent on Hsp70 binding [9]. A recent study has shown that under acute mitochondrial-derived oxidative stress, reversible cysteine oxidation leads to 26S proteasome disassembly, a process that can be reversed by chemical reduction of cysteine residues [11]. For further details on the regulation of the UPS during oxidative stress see [12].

Redox signaling occurs when peroxides operate in redox sensing, signaling and regulation [13]. We hypothesized that cysteine oxidation is a crucial step in the 26S proteasome regulation and its participation redox regulation events. By monitoring the oxidation of mitochondrial and cytosolic peroxiredoxins, as well as peroxiredoxin over oxidation [14] and overall protein thiols in cells [15], we stablished a model of moderate intracellular redox imbalance. We show that even under these conditions the 26S proteasome suffers a cysteine-dependent and disassembly-independent inactivation. We propose an intermediate redox-regulation of the 26S proteasome, with higher relevance during redox signaling events rather than as response to oxidative stress.

## 2 Materials and methods

### 2.1 Chemicals

Hyclone Dulbecco's Modified Eagles Medium/high glucose, (DMEM/high glucose; GE Healthcare), Biochrom Dulbecco's Modified Eagles Medium/low glucose, (DMEM; Biochrom FG0415), Fetal Calf Serum (FCS; Gipco, Fischer Scientific), trypsin-EDTA (Sigma), catalase (Sigma), sodium phosphate (NaPi, Roth), ethylenediaminetetraacetic acid (EDTA, Sigma), diethylenetriaminepentaacetic acid **(**DTPA; Sigma), N-ethylmaleimide (NEM; Sigma Aldrich), Suc-LLVY-AMC (proteasome β − 5 subunit substrate, ENZO), Tris-borate-EDTA buffer (Sigma), BODIPY TMR C5-Maleimide (Invitrogen), antimycin A (Sigma).

### 2.2 Cell culture and treatment

MIN6 cells were cultured in DMEM/high glucose (25 mM) growth medium supplemented with 10% FCS at 37 °C in a humidified 5% CO_2_ atmosphere. Antimycin A (AA) was dissolved (40 mM) in DMSO and a 0.2 mM dilution was freshly prepared in sterile PBS to apply to cell cultures.

For the experiments with palmitate-containing media: sodium palmitate was dissolved in 50% (v/v) ethanol at 70 °C to a final concentration of 200 mM. Pre-warmed DMEM/low glucose (5.5 mM) was supplemented with the indicated glucose concentrations and palmitate was added to a final concentration of 500 µM, 1% FCS and 1% (w/v) FFA-free BSA and was shacked for 2 h at 37 °C. Control cells were treated with 1% FCS, 1% (w/v) FFA-free BSA and 0.12% ethanol.

### 2.3 Immunoblot analysis of peroxiredoxin(s) redox state

The redox state of the cell was evaluated by determining the levels of peroxiredoxin 2 (Prx2, cytosol) and 3 (Prx3, mitochondrial matrix) dimerization, and Prx overoxidation to cysteine sulfinic/sulfonic acid, based on the method described by Cox *et al.* [14]. After treatment with AA or hydrogen peroxide (H_2_O_2_) or palmitate (500 µM during 12 h), the cells were washed twice with ice-cold alkylation buffer (100 mM NaPi, 0.1 mM DTPA pH 7.4, 10 µg/ml catalase and 100 mM NEM; catalase was added 30 min prior to use and the NEM immediately prior to use), lysed with lysis buffer (alkylation buffer+ 0.1% SDS) and incubated at RT for 30 min. The protein content was measured by the DC Protein Assay Lowry (BioRad). Protein extracts were diluted in non-reducing buffer, resolved by SDS-PAGE (15%) and transferred to a nitrocellulose membrane (Whatman®, GE Healthcare, Dassel, Germany) by semi-dry blotting. Membranes were blocked using Odyssey® Blocking Buffer (LI-COR, Bad Homburg, Germany) and diluted 1:5 in PBS. Primary antibodies (rabbit polyclonal anti-peroxiredoxin 3 (Sigma-Aldrich; 1:4000), anti-GAPDH [6C5] mouse monoclonal (abcam; 1:20000), mouse polyclonal anti-peroxiredoxin 2 (Sigma-Aldrich; 1:4000), rabbit polyclonal anti-peroxiredoxin-SO_2/3_ (Abcam; 1:2000) and IRDye^®^ secondary antibodies (LI-COR, Bad Homburg, Germany; 1:20000)) were diluted in Odyssey^®^ Blocking Buffer diluted 1:5 in PBS, containing 0.1% Tween-20. The detection of immunoblots was performed with the Odyssey^®^ imaging system (LI-COR, Bad Homburg, Germany).

### 2.4 Redox fluorescent switch and detection

For the fluorescent labelling of reversibly oxidized protein thiols [15], cells were washed twice with phosphate buffer saline (PBS) and protein extracts were obtained by incubation at room temperature (RT) for 10 min in extraction buffer (50 mM Tris-HCl pH 7.4, 1 mM EDTA, 1% Triton X-100, protease inhibitors and 50 mM NEM; protease inhibitors and NEM were added immediately prior to use). The cell debris was removed by centrifugation and 2% SDS was added to the supernatants, which were incubated for 30 min at 37 °C to block free thiols. The protein content was measured by the DC Protein Assay Lowry and samples were aliquoted and frozen at − 80 °C. Protein extracts (50 μg) were precipitated with 4 volumes of ice-cold acetone, resuspended in 50 µl of TENS buffer (50 mM Tris pH 7.2, 1 mM EDTA, 1% SDS) containing 1 mM DTT and incubated for 10 min at RT. Samples were again precipitated with acetone, resuspended in 50 µl of TENS buffer with 40 μM BODIPY TMR C5-Maleimide and incubated for 30 min at 37 °C. The reaction was stopped by adding 2.5 mM DTT. After precipitating again with acetone and dried at 37 °C the samples were resuspended in 40 µl of 1X non-reducing SDS loading buffer and proteins resolved by SDS-PAGE (10%). After running, gels were washed extensively with double-distilled water and BODIPY fluorescence was detected using a ChemidocTouch^®^ imaging system (BioRad). Total protein was measured by Coomassie staining and detection with the same instrument.

### 2.5 Proteasome activity

The rate of degradation of fluorogenically labeled suc-LLVY-AMC was measured for determining the chymotrypsin-like activity of the β − 5 catalytic subunit located in the 20S core particle [1]. Cells were cultured in 6-well (34.8 mm diameter) plates and treated when reached 80% confluency. After treatment cells were trypsinized, placed in 1.5 ml tubes and washed three times by subsequent centrifugation (2000 ×*g* at 4 °C) and resuspension in ice-cold PBS. Washed cell pellets were resuspended in proteasome lysis buffer (25 mM HEPES, 250 mM sucrose, 20 mM MgCl_2_, 1 mM EDTA, pH 7.4), lysed by three freeze-thaw cycles (liquid nitrogen and 37 °C water bath, respectively). Cell lysates were centrifuged at 14,000 ×*g* for 30´ at 4 °C to remove cell debris. The supernatants were placed in new tubes and the protein content was measured by the DC Protein Assay Lowry. All samples were diluted to a concentration of 1 µg/µl. 10 µl (10 µg) of sample (triplicates) were placed in a black 96-well plate. 90 µl of proteasome activity buffer (150 mM Tris, 30 mM potassium chloride, 7.5 mM MgOAc, 10 mM MgCl_2_, containing either 100 µM ATP for measuring the ATP- stimulated proteolysis, or 15 mM deoxyglucose and 0.1 mg/ml hexokinase for depletion of ATP) was added and incubated during 20´ at RT. The reaction was started by addition of 20 µl of 120 µM suc-LLVY-AMC diluted either in ATP-containing or ATP-depletion buffer. Methyl coumarin liberation was measured with a fluorescence plate reader (λ_ex_= 360 nm, λ_em_= 485 nm). The chymotrypsin-like activity of the proteasome was determined as the rate of methyl coumarin fluorescence increase during the first 30 min of reaction.

### 2.6 Native electrophoresis, in-gel proteasome activity and Western blot

For the separation of 26S and 20S proteasome particles by native PAGE electrophoresis, in-gel chymotrypsin-like activity and detection of β − 5 subunits by Western blot, cell extracts were prepared as for proteasome activity above, with exception that 150 mM cell culture dishes were used. Samples were separated in two aliquots and one was incubated 30 min with 25 mM DTT at 4 °C immediately previous to running the gel. Fifty µg of protein were loaded into non-denaturing 4.5% polyacrylamide gels prepared in proteasome native electrophoresis buffer (10X buffer: Tris-Borate-EDTA 10 × (Sigma) supplemented with 25 mM MgCl_2_) and run during 3 h at 100 V and 4 °C. After running, gels were carefully detached from the glass, washed with proteasome activity buffer and placed into dark boxes for incubation with proteasome activity buffer containing 20 µM suc-LLVY-AMC during 15 min at 37 °C. In-gel methyl coumarin fluorescence was detected using a ChemidocTouch^®^ imaging system. Immediately after scanning, gels were incubated 5 min in PBS containing 0.1% SDS, washed with PBS and proteins were transferred to a nitrocellulose membrane (Whatman®, GE Healthcare, Dassel, Germany) by semi-dry blotting. Protein content was detected by Ponceau S staining and scanning using a ChemidocTouch^®^ imaging system. The detection of β5 subunits by Western blot was performed identically as for peroxiredoxins, but using the Enzo PW8895 primary antibody (1:4000).

### 2.7 Proteomic analysis

A cysteine targeted proteomics approach [15], [16] as performed using the same protein extracts prepared for the redox fluorescent switch. A detailed description can be found under Supplementary Information.

## 3 Results and discussion

### 3.1 Low fluxes of mitochondrial ROS inhibit ATP-stimulated proteasome activity in MIN6 beta-cells

**Fig. 1:**
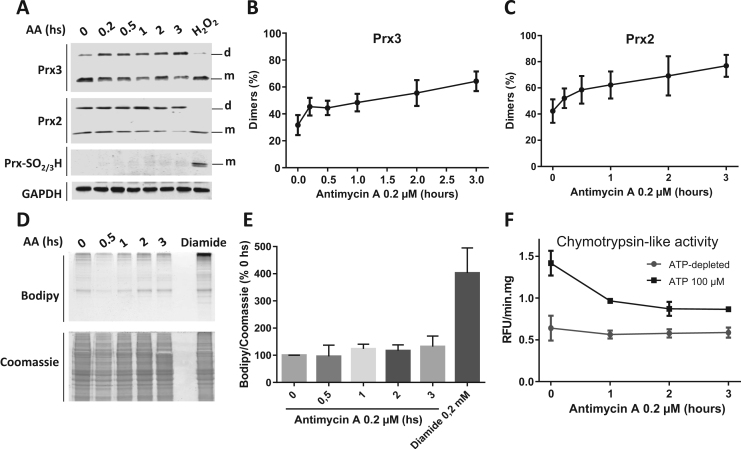
Low fluxes of mitochondrial ROS inhibit ATP-stimulated proteasome activity. A. Dimerization of mitochondrial (matrix) peroxiredoxin 3, cytosolic peroxiredoxin 2 and overoxidation of typical two-cysteine peroxiredoxins to cysteine-sulfinic/sulfonic acid, after incubation of MIN6 beta cells with 0.2 μM antimycin A during the indicated times. A bolus addition of H_2_O_2_ (200 μM, 15 min) was used as a positive control of peroxiredoxin overoxidation. GAPDH was used as a protein loading control (m – monomer; d – dimer). B, C. Quantification of dimeric vs. monomeric Prx3 and Prx2 (n = 4). D. Detection of oxidized protein-cysteine residues labeled with maleimide-BODIPY TMR (top) and total protein stained with coomassie (bottom) under the same conditions as in (A). Formation of disulfide bonds was induced with diamide (0.2 mM, 15 min) as a positive control. E. Quantification of data from D (n = 3). F. ATP-stimulated (black trace) and ATP-independet (grey trace) chymotrypsin-like activity in MIN6 cell lysates upon treatment with antimycin A as in A and D (n = 3).

In order to establish an experimental condition where low fluxes of mitochondrial-derived oxidants are produced without causing a massive oxidation of the cellular components, we tested the dimerization and over oxidation of endogenous peroxiredoxins [14]. The abundance and reactivity of these enzymes make them preferential targets in peroxide-mediated signaling and, therefore, the redox state of these enzymes confers a rapid and extremely sensitive method to evaluate the redox state of the cell and compartments [14], [17]. When MIN6 pancreatic beta cells where exposed to antimycin A (AA, 0.2 µM) to stimulate the production of reactive oxygen species by mitochondrial complex III [18], a time-dependent dimerization of peroxiredoxins 2 and 3 was observed (Fig. 1A-C).

No overoxidation of Prxs to cysteine sulfinic/sulfonic acid (Prx-SO_2/3_H) was observed under these conditions, confirming that only a soft redox imbalance of the cell is induced. A bolus addition of H_2_O_2_ (200 µM, 15 min) was used as a positive control for Prx over oxidation. As expected, this condition also inhibited dimerization of Prxs, as over oxidation occurs upon reaction of a second molecule of oxidant with the sulfenic acid derivative of the catalytic cysteine of the enzyme [14]. We performed the redox fluorescence switch assay to detect overall oxidation of protein cysteine residues [15], without observing significant differences with AA treatment (Fig. 1D and E). Incubation of cells with diamide in order to induce protein disulfide bridges was used as a positive control [15]. No significant accumulation of poly-ubiquitinated proteins was observed under these conditions (data not shown).

However, under the same conditions we observed a time-dependent attenuation of the ATP-stimulated chymotrypsin-like activity of the proteasome (β-5 subunit), while the ATP-independent activity remained unalatered (Fig. 1F). Similar effects were observed when the cells were incubated with palmitic acid (Supplementary Figure 1), a condition that has been shown to induce pancreatic β-cell apoptosis through modulation of Bcl-2 proteins by UPS [19]. The ATP-stimulated activity is representative of the 26S activity, while the ATP-independent activity is more representative of the 20S “core” proteasome activity [9]. This result is not only concordant with previous reports showing a higher susceptibility of the 26S proteasome to inactivation during oxidative stress [20], but suggests that the 26S proteasome could take part in redox signaling processes [21].

### 3.2 Early cysteine-dependent 26S proteasome inhibition is not caused by disassembly

**Fig. 2:**
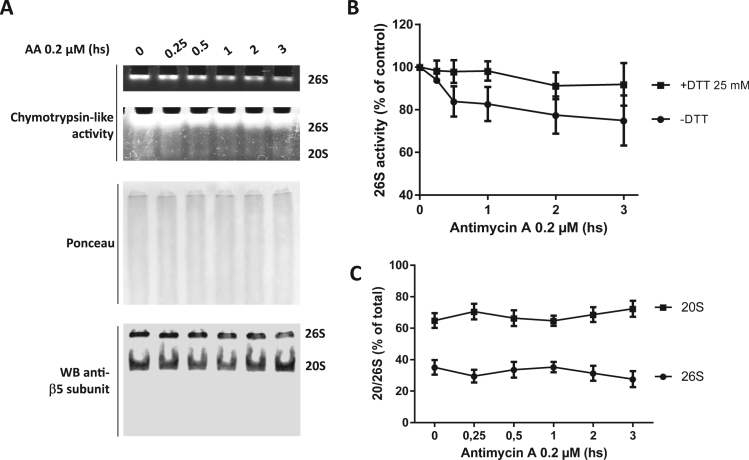
*Low fluxes of ROS induced by antimycin A inhibit the 26*S *proteasome but does not cause disassembly*. A. In-gel chymotrypsin-like activity of proteasomes in MIN6 cells after treatment with antimycin A (0.2 μM) during the indicated times and protein separation by native electrophoresis. The upper band shows the 26S proteasome activity. Due to the much lower 20S proteasome activity a in the lower part of the top panel an overexposed gel is demonstrated showing the faint 20S band and the bright (here not quantifiable 26S band again)(top). Middle: Ponceau staining after protein transfer to a nitrocellulose membrane. Bottom: detection of the β-5 catalytic subunit by Western blot. The 20S and 26S particles (20S + one or two 19S regulatory particles) are indicated in the right side. B. Quantification of the activity shown in A calculated as the fluorescence intensity normalized to Ponceau staining without (circles) or with incubation of the samples with DTT (25 mM, 30 min at 4 °C) prior to sample loading to the gel (n = 3). C. Quantification of β5 catalytic subunit amounts in the 20S and 26S forms shown in A, bottom (n = 3).

In order to confirm that the inhibition observed in Fig. 1F corresponds to the 26S particle inactivation, we performed native gel electrophoresis of MIN6 lysates to separate 26S particles, and measured the in-gel chymotrypsin like activity. Upon incubation of the cells with AA under the same conditions as in Fig. 1, a time-dependent decrease of in-gel chymotrypsin like activity was observed (Fig. 2 A top, and B). Pre-incubation of the samples with DTT (25 mM, 30 min at 4 °C) reversed this inhibition at the first time-points, confirming that the inhibition is dependent on reversible cysteine oxidation (Fig. 2B).

Immunoblot detection of the catalytic β − 5-subunits indicated that in this cell line and under these experimental conditions the proteasome is 60% and 40% under the forms of 20S and 26S particles, respectively (Fig. 2A, bottom). The assembly of the proteasome remained unaffected while the activity suffered a time-dependent inhibition (Fig. 2A and B). This result suggests an intermediate step in the redox regulation of the 26S proteasome during low fluxes of mitochondrial oxidants and redox signaling. It is worth considering that the results shown hereby have been performed using a simplistic pentapeptide substrate for measuring the chymotrypsin-like activity of the proteasome, and that these results may have dramatic effect when considering the activity and selectivity toward complex ubiquitinated protein substrates [5].

In the light of the reversibility of the inhibition by reduction with DTT, we performed a thiol redox proteomics approach, Gelsilox (see supplementary information for full method description), that differentially labels reduced and reversibly oxidized cysteines [15], [16], comparing control samples and cells treated with 0.2 µM AA for 2 h. In label-free experiments we were able to identify several cysteine-containing peptides from proteins belonging to the 26S proteasome (Table 1), while when we performed iTRAQ labelling for more precise quantification, those peptides were not detected even in a targeted approach (results not shown). Few of the Cys-containing peptides identified were reversibly oxidized (Table 1). This was expected taking into consideration that the overall oxidation of protein cysteines within the cell was not increased under this experimental conditions (Fig. 1). Two oxidized cysteine-containing peptides from the 20S particle where identified. According to our results and previous reports [9], [10], [11], these oxidations may not be sufficient to 20S proteasome inactivation, but further unspecific, acute oxidation events. The fact that they appear only in the oxidized form suggest that they could be forming a structural disulfide bond or other form of stable reversible oxidative modification. However, an ATPase and a non-ATPase subunit of the 19S regulator (accession numbers P62334 and Q9D8W5, respectively) were identified via an oxidized cysteine-containing peptide, the latter only identified in the AA-treated samples, and the former identified in both reduced and oxidized form; this suggests that they could form cysteine switches that may reversibly oxidize upon AA treatment. Indeed, a quantitative analysis of the label-free analysis with the MaxQuant software [22] allowed us to estimate that the intensity ratio of the oxidized peptide (AVASQLDCNFLK, accession number P62334 in Table 1) to its protein amount increased upon AA treatment (0.077 for control sample, 0.141 for AA-treated sample; ratio of the peptide intensity vs the protein intensity in each sample). Interestingly, the oxidized cysteine residue from the ATPase regulatory subunit 10B is located only 11 amino acids distant from the ATP binding-site [23], which suggests a functional implication of this oxidation, although it is not possible to confirm a direct implication so far. Further functional and quantitative proteomics studies are needed in order to determine whether these subunits are the main responsible for a regulated, specific regulation of the 26S proteasome during redox signaling.

**Table 1:** Proteasome cysteine-containing peptides identified upon antimycin treatment.

**Accession**	**Description**	**Peptide Sequence**	**Control**	**Antimycin**
P62334	26S protease regulatory subunit 10B	AVASQLD**C**NFLK	R; O	R; O
P62192	26S protease regulatory subunit 4	AI**C**TEAGLMALR	R	ND
Q3TXS7	26S proteasome non-ATPase regulatory subunit 1	MEEADALIESL**C**R	R	R
		VLSMTET**C**R	R	R
Q8BG32	26S proteasome non-ATPase regulatory subunit 11	TTANAIY**C**PPK	R	ND
Q9D8W5	26S proteasome non-ATPase regulatory subunit 12	AIYDTP**C**IQAESDK	ND	O
Q99JI4	26S proteasome non-ATPase regulatory subunit 6	AEYL**C**QIGDK	R	ND
Q9R1P0	Proteasome subunit alpha type−4	AT**C**IGNNSAAVSMLK	O	O
Q9QUM9	Proteasome subunit alpha type−6	D**C**AVIVTQK	O	O
		YGYEIPVDML**C**K	R	R

R, reduced; O, oxidized, ND, not determined, grey-highlights, reversibly oxidized cysteine residues.

## 4 Conclusions

The 26S proteasome is highly susceptible to oxidative inactivation whereas the 20S proteasome undergoes activation during oxidative stress, allowing the degradation of oxidized proteins [9]. The mechanism proposed so far for the oxidative inactivation of the 26S proteasome involves particle disassembly into its 20S “core” catalytic and 19S regulatory subunits [11]. We show that the 26S proteasome is sufficiently sensitive to be redox regulated even during formation of low fluxes of oxidants within the cell. Given the experimental conditions established herein, and the fact that peroxiredoxins are preferential targets for peroxide-induced signaling [17], it is possible to speculate that redox regulation of the 26S proteasome may occur via a thiol peroxidase sensor [24], [25].

**Fig. 3:**
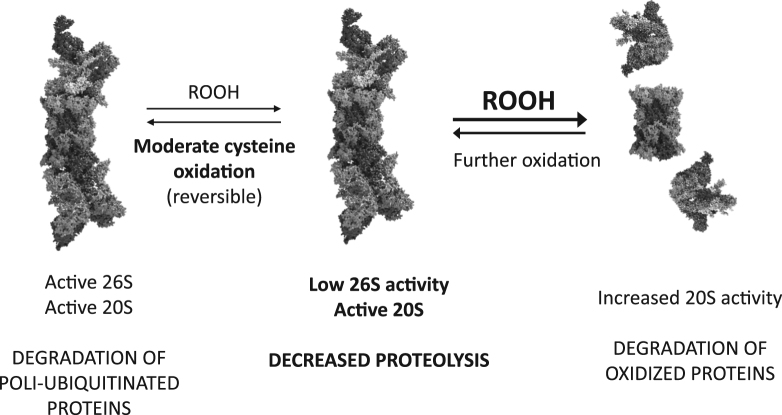
*Cysteine oxidation-mediated regulation of proteasome activity during redox signaling and stress.* Low fluxes of mitochondrial-derived peroxides associated with redox signaling processes cause moderate and site-specific oxidation of cysteine residues, leading to a transient, reversible inactivation of the 26S activity. Degradation of poly-ubiquitinated proteins is thereby blocked during while this redox signal lasts. Alternatively, under conditions of oxidative stress 19S particles are released, thus generating free, open 20S proteasomes [10] that contribute to the degradation of oxidized proteins generated during the stress. The 3D structures shown are only for illustrative purposes and were taken from [21].

Here we show that even during low fluxes of mitochondrial-derived reactive oxygen species formation, cysteine oxidation could mediate the inhibition of the 26S proteasomes, involving inactivation of the 19S regulatory subunit while still interacting with the 20S core particle (Fig. 3), providing an intermediate step in the redox regulation of proteolysis, with special relevance in redox signaling conditions rather than under oxidative stress.

## Appendix A Supporting information

Figure S1 *Palmitate induces a redox imbalance and attenuated 26S proteasome activity*. A. Dimerization of mitochondrial (matrix) peroxiredoxin 3, after incubation of MIN6 beta cells with palmitate (PA, 500 μM, 12 h) in the presence of different concentrations of glucose (5.5, 15, 25 mM). GAPDH was detected as a protein load control. B. Quantification of results shown in A, as means of percentage of peroxiredoxin 3 in the dimeric form. C. ATP-stimulated chymotrypsin-like activity in MIN6 cell lysates upon treatment with palmitate (500 μM) during the indicated times (n = 3).
