# Supplementary figures and images for: Early cysteine-dependent inactivation of 26S proteasomes does not involve particle disassembly
Source: Redox Biol. 2018 Feb 22;16:123–8. doi: 10.1016/j.redox.2018.02.016 (PMC5952582; doi:10.1016/j.redox.2018.02.016)

## Slide 1
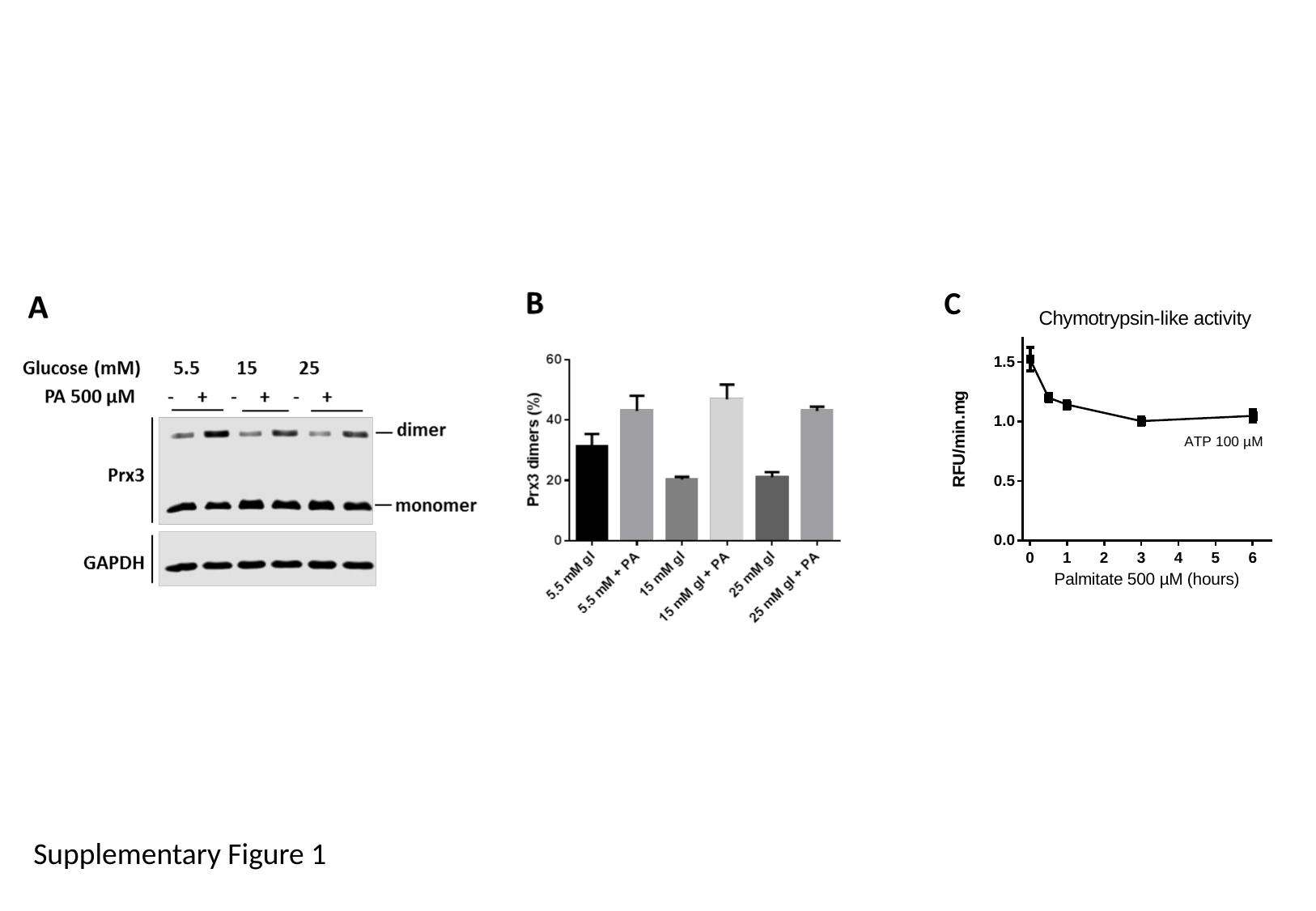

C
Supplementary Figure 1

Supplement: Supplementary file 1 — Figure S1 Palmitate induces a redox imbalance and attenuated 26S proteasome activity. A. Dimerization of mitochondrial (matrix) peroxiredoxin 3, after incubation of MIN6 beta cells with palmitate (PA, 500 μM, 12 h) in the presence of different concentrations of glucose (5.5, 15, 25 mM). GAPDH was detected as a protein load control. B. Quantification of results shown in A, as means of percentage of peroxiredoxin 3 in the dimeric form. C. ATP-stimulated chymotrypsin-like activity in MIN6 cell lysates upon treatment with palmitate (500 μM) during the indicated times (n = 3). [file mmc1.pptx]
