# Supplementary material for: Early cysteine-dependent inactivation of 26S proteasomes does not involve particle disassembly
Source: Redox Biol. 2018 Feb 22;16:123–8. doi: 10.1016/j.redox.2018.02.016 (PMC5952582; doi:10.1016/j.redox.2018.02.016)
Supplement: Supplementary file 2 — Supplementary material. [file mmc2.docx]

***Proteomic analysis***

*In-gel protein digestion (stacking gel)*: 80 µg of the same protein extracts prepared for the redox fluorescent switch were suspended in a volume up to 50 μL of sample buffer, and then applied onto 1.2-cm wide wells of a conventional SDS-PAGE gel (1.5 mm-thick, 4% stacking, and 10% resolving). Electrophoretic run was stopped as soon as the front entered 3 mm into the resolving gel, so that the whole proteome became concentrated in the stacking/resolving gel interface. The unseparated protein bands were visualized by Coomassie staining, excised, cut into cubes (2 x 2 mm), and placed in 0.5 ml microcentrifuge tubes (1). The gel pieces were destained in acetonitrile:water (ACN:H_2_O, 1:1), reversibly oxidized cysteine residues were reduced with 10 mM DTT for 1 h at 56 ºC and then alkylated with 50 mM iodoacetamide for 1 h at room temperature in darkness, and proteins were digested *in situ* with sequencing grade trypsin (Promega, Madison, WI) as described by Shevchenko *et al.* (2) with minor modifications. The gel pieces were shrunk by removing all liquid using sufficient ACN. Acetonitrile was pipetted out and the gel pieces were dried in a speedvac. The dried gel pieces were re-swollen in 50 mM ammonium bicarbonate pH 8.8 with 60 ng/mL trypsin at 5:1 protein:trypsin (w/w) ratio*.* The tubes were kept in ice for 2 h and incubated at 37°C for 12 h. Digestion was stopped by the addition of 1% TFA. Whole supernatants were dried down and then desalted onto OMIX Pipette tips C18 (Agilent Technologies) until the mass spectrometric analysis.

*Reverse phase-liquid chromatography RP-LC-MS/MS analysis*: the desalted protein digest was dried, resuspended in 10 μL of 0.1% formic acid and analyzed by RP-LC-MS/MS in an Easy-nLC II system coupled to an ion trap LTQ-Orbitrap-Velos-Pro hybrid mass spectrometer (Thermo Scientific). The peptides were concentrated (on-line) by reverse phase chromatography using a 0.1mm × 20 mm C18 RP precolumn (Thermo Scientific), and then separated using a 0.075mm x 250 mm C18 RP column (Thermo Scientific) operating at 0.3 μl/min. Peptides were eluted using a 180-min dual gradient from 5 to 25% solvent B in 135 min followed by gradient from 25 to 40% solvent B over 180 min (Solvent A: 0,1% formic acid in water, solvent B: 0,1% formic acid, 80% acetonitrile in water). ESI ionization was done using a Nano-bore emitters Stainless Steel ID 30 μm (Proxeon) interface. The Orbitrap resolution was set at 30,000. Peptides were detected in survey scans from 400 to 1600 amu (1 μscan), followed by twenty data dependent MS/MS scans (Top 20), using an isolation width of 2 u (in mass-to-charge ratio units), normalized collision energy of 35%, and dynamic exclusion applied during 30 seconds periods.

*Data analysis:* Peptide identification from raw data was carried out using the SEQUEST algorithm (Proteome Discoverer 1.4, Thermo Scientific). Database search was performed against uniprot-MusMusculus.fasta. The following constraints were used for the searches: tryptic cleavage after Arg and Lys, up to two missed cleavage sites, and tolerances of 20 ppm for precursor ions and 0.8 Da for MS/MS fragment ions and the searches were performed allowing optional Met oxidation, Cys carbamidomethylation and Cys addition of N-ethylmaleimide. Search against decoy database (integrated decoy approach) using false discovery rate (FDR) < 0.01.

*Quantitative data analysis:* Peptide identification from raw data was carried out using MaxQuant 1.6.0.16, a quantitative proteomics software package used for analyzing large-scale mass-spectrometric data sets (3). Database search was performed against uniprot-MusMusculus.fasta. The following constraints were used for searches: Standard type of analysis, trypsin/P digestion, up to two missed cleavage sites allowing optional Met oxidation, Cys carbamidomethylation and Cys addition of N-ethylmaleimide. Protein label-free quantification was performed excluding Cys-modified peptides and with a minimum ratio count of 2. Search against decoy database (integrated decoy approach) using false discovery rate (FDR) < 0.01. Quantification of individual peptides was corrected against the quantification of the protein to which they belong by dividing the peptide intensity value by the protein intensity value.

References

1. Moreno, M. L., Escobar, J., Izquierdo-Alvarez, A., Gil, A., Perez, S., Pereda, J., Zapico, I., Vento, M., Sabater, L., Marina, A., Martinez-Ruiz, A., and Sastre, J. (2014) Disulfide stress: a novel type of oxidative stress in acute pancreatitis. *Free Radic Biol Med* **70**, 265-277

2. Shevchenko, A., Wilm, M., Vorm, O., and Mann, M. (1996) Mass spectrometric sequencing of proteins silver-stained polyacrylamide gels. *Anal Chem* **68**, 850-858

3. Tyanova, S., Temu, T., and Cox, J. (2016) The MaxQuant computational platform for mass spectrometry-based shotgun proteomics. *Nat Protoc* **11**, 2301-2319
